# Music therapy in stress: proposal of extension to Assisted Reproduction

**DOI:** 10.5935/1518-0557.20140006

**Published:** 2014

**Authors:** Eliamar Aparecida de B. Fleury, Mario S. Approbato, Tatiana M. da Silva, Monica Canedo S. Maia

**Affiliations:** 1 Federal University of the State of Goiás (UFG). Laboratory of Human Reproduction of Hospital das Clínicas - Goiânia (GO) - Brazil

**Keywords:** age, sperm parameters, DNA fragmentation, sperm quality, MSOME

## Abstract

Infertility is recognized as a disease of several causes by the World Health Organization. Assisted reproduction has a historic of progress since the development of biotechnology. Women who need this treatment are under stress and emotional tension. Since the 1980´s, professionals have been worried with psychological aspects of the patient that undergoes the Assisted Reproduction treatment. Studies about music therapy show positive results in diminishing/reducing patients’ emotional stress in many specialties. However, there were not studies showing scientific results of this therapeutic approach. There were, though, works in correlated areas, such as Gynecology and Obstetrics. Therefore, a literature review was conducted in the following data bases: PubMed, Lilacs, Medline and Scielo in studies published between 2004 and January 2014. The descriptors/key words used were music therapy and stress; music therapy and infertility; music therapy and assisted reproduction; music therapy and gynecology and obstetrics. The therms were searched both in Portuguese and English. Therefore, this study aims to analyze what is discussed in literature about music therapy and stress in the context of health. The study will be a base for further research in the post-graduation program about the use of music therapy on emotional stress experienced by women during assisted reproduction. Articles about correlated themes, such as gynecology and obstetrics, were also considered, trying to relate them to assisted reproduction.

## INTRODUCTION

The use of music as a therapeutic agent dates from centuries ago. There are historic reports of this use in Sumer, Babylon, and Egypt. Ancient Greece is considered the precursor of contemporary music therapy, with the studies of Pythagoras, Plato, and Aristotle, which somehow contributed for the scientific foundations of music therapy nowadays (Prinou, 1993). In the United States, in the end of the 18th century, two articles are published about the use of music with/ for therapeutic purposes. In the beginning of the XIX century, Samuel Mathews and Edward Atlee, then medical students at University of Pennsylvania, wrote thesis about the use of music in treatment of diseases (Maranto, 1993). The first documented/recorded clinical use of music therapy in an institution dates of 1832, followed of other studies with the goal of establishing a philosophical and historical bases for music therapy. In 1903, the National Therapeutic Society of New York was founded, and in 1913, Music and Health, the first Music Therapy Journal was published. In 1919, at Columbia University the first course of Music Therapy was taught. In 1926, the National Association for Music in Hospitals was founded, and in 1941 the National Foundation for Music Therapy was founded. In 1944, the first program for specific training for music therapists was created at Michigan State University. In 1971 a second Music Therapy association was created/founded, the Urban Federation for Music Therapists (UFMT). Later, in 1975, its name was changed to American Association for Music Therapy (Maranto, 1993). In many countries in Europe there is a history if development of music therapy. In Austria, the interest for music therapy started in the end of the 1950s. In 1954, the Austrian Society for the Promotion of Music Therapy was founded, with the objective of supervising the scientific use of music therapy (Halmer-Stein *et al*., 1993). In Denmark the first music therapy association was founded in 1969 (Danish Society for Music Therapy). In the same year, the first Danish music therapy magazine was published, and in 1982 the first Music Therapy training program started, at Aalborg University (Kortegaard & Pedersen, 1993). In Finland, the first publication about Music Therapy was in 1960, followed by other studies (Lehtonen, 1993). Music Therapy also developed in other countries, such as Korea (Heui, 1993), France (Lecourt, 1993), Italy (Di Franco & Perilli, 1993), Hungary (Konta & Varga, 1993), Greece (Prinou, 1993), Mexico (Polit, 1993), Puerto Rico (Colon, 1993); Spain (Blasco & Vicente, 1993), England, Ireland, Lithuania, Portugal, Sweden, Australia, New Zeland, Israel and Japan (Barcellos, 2012), among other countries. In South America, the movement of music therapy started in Argentina, with its clinical use and formal recognition as a subject in 1948 (Wagner & Benenzon, 1993). Since then, music therapy has been spread in countries such as Brazil (Barcellos & Santos, 1993), Uruguay (Florez *et al*., 1993), Bolivia, Chile, and Colombia (Barcellos, 2012).

In Brazil, music therapy started in the field of psychiatry, in 1955. The first two Music Therapy Associations were founded in 1968, in the states of Rio de Janeiro and Rio Grande do Sul (Costa, 2006). In the year 1969 the first academic training was created, as a Post-Graduation course in the state of Paraná (Barcellos & Santos, 1993).

In 1972, in the state of Rio de Janeiro, a technical course was created to form certificated professionals in music therapy. Later on, in 1973, after due modifications, this course was recognized as a 4 year-graduation course (Costa, 2006). The beginning of the movement in the state of Goiás occurred in 1990, with the foundation of the Music Therapy Society of the State of Goiás (Sociedade Goiana de Musicoterapia, in Portuguese) (Barcellos & Santos, 1993), currently Music Therapy Association of the State of Goiás. In this state, starting in 1993, three post-graduation classes were offered at Federal University of Goiás. In 1999, the graduation course started being offered, this being the first graduation course offered by a federal university in Brazil (Zanini, 2006). Nowadays many Brazilian states offer Graduation and/or Post-Graduation courses and have class associations/professional associations (Barcellos & Santos, 1993). In 1996, it was created the Brazilian Union of Music Therapy Associations (UBAM, in its Portuguese initials) (Conde & Ferrari, 2008). In spite of the development showed, there still is a preconceived idea that this therapy is restricted to listening recorded songs, in a receptive attitude of the client (Zanini, 2009). However, the use of music in medicine is different from music therapy in medicine (Bradt *et al*., 2013). The first is an intervention realized by a health professional that is not graduated in music therapy, and the intervention is by musical audition, may it be with the use of recorded or live music. The second refers to interventions conducted by a professional with qualifications/ graduated in music therapy (Bradt *et al*., 2013), with a degree or post degree, and the treatment performed by means of sound-musical link (UBAM, 2010). It is relevant to understand the particularities between these approaches, both of them being an important form of action towards the patient through the music (Bradt *et al*., 2013). In music therapy applied to medicine, the music and the relation client-therapist are necessary and used likewise to treat the client´s biomedical and/or psychosocial needs, helping the patient to deal with or to cope with the health condition (Bruscia, 2000). Music therapy is defined as “the use of music and/or its elements (sound, rhythm, melody, and harmony) by a qualified music therapist, with a client or group, in a process to facilitate and promote the communication, relation, learning, mobilization, expression, organization, and other relevant therapeutic objectives, in the sense of reaching physical, emotional, mental, social, and cognitive needs. Music therapy aims to develop potentials and/or restore functions of the individual so that he/she may reach a better intrapersonal and/or interpersonal integration, and as a consequence, a better quality of life, by prevention, rehabilitation or treatment” (UBAM, 1996). Therefore, music therapy is a self-expressive therapy that uses the music as the main intervention element, considering the presence of the verbal in the relation music therapist-patient. A broad spectrum of scientific studies shows the benefits of music therapy with patientes in different treatments and medical specialties. However, there were not found studies about the use/ of music therapy with women undergoing assisted reproduction. Therefore, this article aims to review the literature, listing the benefits of music therapy with patients in other correspondent themes.

## MATERIALS AND METHODS

The articles researched were those published in data bases PubMed, Lilacs, Medline and Scielo between the years of 2004 and January 2014. The descriptors/ key words used were music therapy and stress; music therapy and infertility; music therapy and assisted reproduction; music therapy and gynecology and obstetrics. The therms were searched both in Portuguese and English.

The criteria of inclusion were: articles published in Portuguese or English between the years of 2004 and January 2014; articles with key words pre-established in the titles and/or abstract; researches conducted with human beings. The criteria of exclusion were: articles that do not have the pre-established key words in the title and/or abstract; researches conducted with children, adolescents, individuals with dementia; researches with animals; and researches with association of different kind of non- pharmacological therapeutic approaches. The articles were preselected after the reading of the titles and abstracts. After the articles that fit the criteria were identified, the articles were read throughout.

## RESULTS

A total of 311 articles were found, and 22 were selected based on pre-established criteria of inclusion. Other bibliographies were consulted when necessary, based on references from the analyzed articles.

## DISCUSSION

### Infertility, Assisted Reproduction and Stress

Infertility is recognized as a disease by World Health Organization (WHO). It is registered in the International Classification of Diseases - ICD - 10 (Freitas *et al*., 2008). It is a public health problem that affects 8% to 15% of couples worldwide (Brazilian Ministry of Health, 2013). It is a disease of the reproductive system defined by the failure to achieve a clinical pregnancy after 12 months or more of regular unprotected sexual intercourse. Primary infertility is when a couple is unable to conceive even with regular unprotected sexual intercourse. When there is a failure in the conception after previous pregnancies it would be classified as secondary infertility (WHO, 2014).

There are many causes for infertility, such as sexually transmitted diseases, anovulation, endometriosis, hyperprolactinemia, thyroid diseases, sedentary lifestyle, smoking, illegal drugs, exposure to harmful environmental or work factors (radiation, chemical substances), sociocultural factors (stress, excessive physical activity and diet), and age (Ribeiro, 2007).

Other factors are several abortions (Andrade *et al*., 2010), family history of endometriosis (Giudice, 2010), anorexia, uppurative appendicitis, tubal disease, luteal phase defect, (Lopes & Donadio, 1997), cervical factor (Ribeiro *et al*., 2000), polycystic ovary syndrome, immunological factors (Badalotti *et al*., 1997) and major hormonal disorders. There are also factores related to male infertility (Chachamovich, 2009).

Since the first successful pregnancy by in vitro fertilization (IVF) in the United States in 1981, there was a growing use of assisted reproduction technology in infertility treatment/ treatment of infertility. Between the years of 1997 and 2009, the use of IVF rose 84%. In 2009, 101.090 cycles of non-frozen embryos were carried out (Beall & DeCherney, 2012).

In Brazil, the National Health Care Politics for Human Assisted Reproduction establishes the support of the National Health System (SUS) for the treatment of infertility. It is offered in University Hospitals (teaching hospitals) and public hospitals (Brazilian Ministry of Health, 2013). The Human Assisted Reproduction (HAR) consists in a group of techniques composed by: programed sexual intercourse, artificial intrauterine insemination, and in vitro fertilization (classical in vitro fertilization and in vitro fertilization by intracytoplasmic sperm injection). A multidisciplinary team evaluates the follicular development, the detection and induction of the ovulation, as well as the encounter of the gametes and the optimization of the luteal phase (Freitas *et al*., 2008).

From the emotional point of view, infertility can be understood as a threatening situation, and it can cause many different sensations and feelings (Melamed, 2013). Nowadays, infertility is recognized as a peculiar disease, characterized by invisibility and the possibility of no physical discomfort (Straube, 2013). There are evidences that the infertility should not be understood as a classical disease, with its components of pain, life risk, and hospitalization, because it can exist without these components, although infertility generates many psychological alterations. The peculiarity of infertility is linked to the desire to procreate, making the experience one of the most stressful in a couple or individual´s life (Straube, 2013). Even if the cause of infertility is related to the man, the woman feels it with more intensity (Melamed, 2006).

The experience of infertility can cause harm to the person, with loss of self esteem, fear, alienation, social isolation, loss of social status, and in some cases, violence (Souza, 2008), severe negative reflections in women´s life quality (Chachamovich, 2009). The experience of infertility, and assisted reproduction treatment (ART) have been associated to harmful psychological and social consequences for the individual (Watkins & Baldo, 2004; Cousineau & Domar, 2007).

A cross-sectional study included 1406 couples who were consecutively referred patients undergoing fertility treatments in Denmark in the year 2000. A total of 1049 men and 1131 women were included in the study. Among these, 11,6% of the women and 4,3% of the men showed severe symptoms of depression significantly associated to the increase of anguish and distress related to the infertility, regarding both the individual and the couple (Peterson *et al*., 2014).

Different biological mechanisms related or triggered by stress can diminish the fertility. However, it is very hard to establish cause-effect relationship between this disease and stress, due to the multiplicity of causes of infertility, including male causes (Moreira, 2010). Yet, the diagnosis of infertility and the assisted reproduction treatment are evidenced in literature as stress-causing agents. It´s necessary, then, for these patients, care beyond medical aspects (Lopes, 2013), taking into consideration emotional factors that are involved (Peterson, 2014), and the music therapy can be included in these interventions.

### Music and Health

Since ancient times, music, which is an art present in all cultures (Rojas, 2011), has been associated to disease treatment. It has been used by diferent peoples to heal. However, the study of music for a systematic use in disease treatment occurred in 1880, by Dogiel, in França (Standley, 1986).

Since then, a considerable number of studies report the physiological and psychological effects of music in human organism and in different medical procedure, favoring, among other things, breath control, drugs dosage reduction, relaxation of the pregnant woman during and after delivery (Livingston, 1979), and reducing anxiety of patients on invasive mechanical ventilation (Naváis, 2013). The use of music also favors the decrease of anxiety, pain and use of drugs in postoperative patients (Nilsson *et al*., 2003); distraction, movement reduction, psychological trauma reduction, and optimization of cicatrization in patients with severe burns (Prensner *et al*., 2001).

A randomized clinical trial was conducted with experimental and control groups. The goal was to investigate the effect of music, the effect of relaxation techniques, and the combination of both in situation of pain after gynecological surgery, with 311 patients with ages from 18 to 70 years. The results showed that the intervention groups had significantly less post-test pain than control group in postoperative days 1 and 2, respectively (P = 0.0220.001). The individuals who received the interventions and patient-controlled analgesia had from 9% to 29% less pain than the controls that used only analgesia (Good *et al*., 2002).

Other benefits of the use of music are also related, such as alteration of beta-endorphin levels; regarding psychological aspects, the reduction of fear, and in care of cardiac patients (Vollert *et al*., 2003). The use of music provides relaxation for Alzheimer´s disease patients undergoing treatment (Kumar *et al*., 1999). It is also used during gynecological or andrological exams that cause embarrassment for the patient (Lee *et al*., 2002). The anxiolytic effect of music has been studies by many medical areas, including cardiology, radiology, pulmonology, and gastroenterology (Rojas, 2011).

Studies show the influence of music in plasma levels of hormones, such as the adrenocorticotropic hormone (ACTH) and the cortisol (CORT) (Miluk-Kolasa *et al*., 1994), which are responsible for cardiac frequency alteration, systolic pressure and emotional state linked to stress, as well as hypophysis hormones, such as growth hormone (GH) and prolactin (PRL) (Gerra *et al*., 1998), and in the levels of epinephrine (EPI), norepinephrine (NE), serotonin, melatonin (Kumar *et al*., 1999) and beta-endorphin (Gerra *et al*., 1998).

In the neurophysiological aspect, music induces to relaxation (Nilsson, 2009) due to its impact in the central nervous system automatized/automated responses (Gillen 2008; Bradt *et al*., 2013). It is believed that the music anxiolytics effect is obtained through its suppressive action on the sympathetic nervous system, leading to the decrease of adrenergic activity, and decrease of neuromuscular excitement (Chlan 1998; Gillen 2008; Bradt *et al*., 2013). It is considered, though, that personality factors and individual differences in musical selection can mediate such effects (Chanda & Levitin, 2013).

### Music therapy and Health

Music therapy is a non-pharmacological, self expression therapy that uses music as main element of intervention. Its application in health ambit has shown positive results for people with neurological problems (Schlesinger *et al*., 2009; Correia, 2010); cardiac patients (Hanser & Mandel, 2005; Zanini, 2009; Hanser, 2014), and cancer patients (Li *et al*., 2011; Lin *et al*., 2011; Alcântara-Silva, 2012).

In investigations about music therapy during endoscopic procedures, the results show that music therapy intervention leaded to smaller use of anxilolytics (Lee *et al*., 2002; Andrada *et al*., 2004). The music therapy approach has also been useful for health professionals, resulting in a significative statistic decrease (variation = - 60%, P<0.001) in stress level after the music therapy program (Taets, 2013).

Interventions in music therapy always involve the patient in any kind of musical experience, based on the four basic methods, according to Bruscia (2000): musical improvisation, re-creation, musical composition, and receptive methods (receptive experiences). In musical improvisation methods, the client improvises and makes his own music, playing an instrument or singing. In re-creation, the patient plays or sings, musically reproducing a model presented before. In musical composition the client writes songs, lyrics, instrumental pieces, or even any type of musical material (e.g. videos, audio tapes) with the technical support of the music therapist. In the receptive methods, recorded songs or live songs are used, and then the patient answers to the experience in different ways: silently, verbally, or by means of another expressive modality (Bruscia, 2000). Therefore, the many ways by which human beings get involved with and relate themselves to music are music therapy basic tools. Whichever the kind of musical experience chosen for interventions, the patient will be engaged in the activity, always with therapeutic purposes (Bruscia, 2000). It is important to emphasize that the musical experience is unique for each person, and the music anxiolytic effects are determined by the musical taste of the subject and his individual inclinations (Rojas, 2011; Bradt *et al*., 2013), so the sound and musical history (Barcellos, 1999) of the subject is highly significative in the direction of music therapy interventions.

### Music therapy, Music and Stress

A randomized clinical trial was conducted with 60 elective surgery patients, with the objective to investigate the effects of receptive music therapy in stress reduction. The trial showed that the levels of cortisol in plasma decreased during the surgery in both of the groups of patients who listened to music and it rose in control group. The three groups were equivalent regarding sex, age, surgery indication, and type of surgery and anesthesia. The level of cortisol was measured by fluorescence polarization immunoassay. Other studies about the effect of music therapy in postoperative acute psychological stress showed reduction in the levels of salivary or urinary cortisol after music therapy, similar to this finding (Leardi et. al., 2007).

Raglio *et al*. (2012) investigated the alterations relation in cardiac physiological parameters, such as heart rate, QT interval and its variability and alterations in psychological parameter, in only one music therapy session of 30 minutes, with active experience, through musical improvisation with rhythmic and melodic instruments. The study was conducted with 8 individuals (4 men and 4 women) apparently healthy and with ages from 26 to 51 years. Results showed that the mean of cardiac frequency was reduced during the music therapy session, occurring the increase in only one case during session. Parameters in variability time domain of cardiac frequency were not affected by the music therapy session. The alterations in the psychological parameters were evaluated through the analysis of audio and visual recording of the session, according to the intersubjective theory. For the analysis of the subjects and the music therapist’s emotional behaviors and association to sound parameters (usually associated to changes in emotional conditions), a music therapy checklist was used (Raglio *et al*., 2007). Although the sample was small, the results suggest that the evaluation of the cardiac and psychological answer to the music therapy is viable. This indicates that this methodological approach can be used to test the benefits of music therapy in different clinical conditions (Raglio *et al*., 2012).

Jiménez-Jiménez *et al*. (2013) conducted a randomized, double blind clinical trial, with parallel groups, with 40 patients between 27 to 70 years old, equally divided into experimental and control groups. The patients underwent varicose veins surgery and received music receptive intervention. The authors showed that the anxiety state, and punctuation in tension sensation scale after the surgery were significantly inferior in the group that received musical intervention the results suggest that the receptive musical intervention is effective in the diminution of anxiety and stress in subjects who underwent this kind of surgery. On the other hand, in a study conducted with 40 patients with ages from 40 to 60 who underwent laparoscopic surgery or cholecystectomy under general anesthesia, randomly assigned to listen to music or not during surgery, the results showed that although the patients who had listened to music reported feeling less pain, the difference was not statistically meaningful. The mean arterial pressure was slightly higher in patients who listened to music than in those who did not. Although previous works suggested that music reduces the preoperative stress and can be useful during sedation, the results found in this study did not show the validity of the use of music during surgery (Szmuk *et al*., 2008).

An experimental randomized controlled study with pre and post test was conducted in Taiwan with 236 pregnant women. They were divided into two groups: control (n=120) and experimental (n=116), with musical audition interventions during 30 minutes, at home environment, listening to pre chosen songs. The study showed that the anxiety, psychological stress, and depression decreased (Chang *et al*., 2008). Bauer *et al*. (2010) conducted a duble-blind study with 61 pregnant women hospitalized due to many high risk obstetric problems. The authors concluded that music therapy interventions were effective in stress relief during pre delivery with pregnant women who were mildly to moderately afflict.

An intervention research was conducted with pre and post test in a single group, with 34 female volunteers from different professional areas of a private hospital in Rio de Janeiro (Brazil). The researched demonstrated that the music therapy was effective in reducing the daily stress, with a statistic reduction (P<0.01) in the subjects self perception (Taets, 2013).

A study with the purpose to investigate the effects of music therapy in pain and salivary cortisol levels in patient who had undergone colonoscopy was conducted with 29 patients. These patients were randomly divided into two groups during the exam: musical audition group (n = 15); and a group without the music (n = 14). After the exam, the patients were invited to classify the pain felt during the procedure. Those patients who listened to music showed smaller scores of pain. The salivary levels had a significantly smaller increase in the group that listened to music. The study concluded that music therapy during colonoscopy reduces the stress that is related to fear, as indicated by alterations in salivary cortisol levels (Uedo *et al*., 2004). A research with pre and post test with experimental and control groups was conducted with the objective to identify the influence of music therapy in perceived stressors and anxiety levels of 100 hemodialysis patients. The data were analyzed by t test and chi-square test. The results confirmed that music therapy positively influenced the reduction in self perceived stress and anxiety levels. The scores on State-Trait Anxiety Inventory tests showed positive, statistically relevant results, demonstrating the validity of using music therapy with hemodialysis patients (Cantekin & Tan, 2013).

### Music Therapy in Assisted Reproduction

In spite of the extensive literature review, articles about music therapy in assisted reproduction were not found. It is necessary, therefore, the realizations of studies about this theme.

## FINAL CONSIDERATIONS

Music therapy is a supporting therapy for medical practice in different specialties. We believe that the stress caused by the assisted reproduction treatment can be mitigated by music therapy with specific methods. We consider that the existing researches offer a starting point for us to explore the possible results of the music therapy with this target group. There is an intervention project underway in music therapy in assisted reproduction, at the Laboratory of Human Reproduction of the Federal University of Goiás.
